# Chronic pain after primary total and medial unicompartmental knee arthroplasty for osteoarthritis: a Danish nationwide cross-sectional survey

**DOI:** 10.2340/17453674.2025.44898

**Published:** 2025-10-27

**Authors:** Jens LAIGAARD, Saber M ALJUBOORI, Lone NIKOLAJSEN, Ole MATHIESEN, Troels H LUNN, Martin LINDBERG-LARSEN, Søren OVERGAARD

**Affiliations:** 1 Department of Orthopaedic Surgery and Traumatology, Bispebjerg University Hospital; 2 Department of Clinical Medicine, University of Copenhagen; 3 Department of Clinical Medicine, Faculty of Health, Aarhus University; 4 Department of Anaesthesiology and Intensive Care, Aarhus University Hospital; 5 Centre for Anaesthesiological Research, Department of Anaesthesiology, Zealand University Hospital Køge; 6 Department of of Anaesthesia and Intensive Care, Copenhagen University Hospital, Bispebjerg and Frederiksberg; 7 Department of Orthopaedic Surgery and Traumatology, Odense University Hospital, Denmark

## Abstract

**Background and purpose:**

Contemporary data on the risk of chronic pain after total knee arthroplasty (TKA) and unicompartmental knee arthroplasty (UKA) is limited. Therefore, we aimed to investigate the incidence of chronic pain, pain characteristics, patterns of analgesic use, patient satisfaction, and willingness to undergo the same surgery again, 1 year after primary TKA and UKA for osteoarthritis.

**Methods:**

We conducted a nationwide online survey among unselected patients who underwent primary TKA or medial UKA for primary osteoarthritis in Denmark. At 1 year postoperatively, we assessed the incidence of moderate to severe pain (≥ 4 on the 0–10 numerical rating scale), frequency of pain, pain interference with everyday life, the Western Ontario and McMaster Universities Osteoarthritis Index (WOMAC) pain domain, the Douleur Neuropathique 4 interview (DN4i), use of analgesics, satisfaction, and willingness to undergo the same surgery again.

**Results:**

We sent survey invitations to 2,580 TKA patients and 1,007 UKA patients who underwent surgery in 2022. Of the 70% TKA respondents, 25% had moderate to severe chronic pain, 82% were satisfied/very satisfied with the result of surgery, and 86% indicated that they would choose to undergo surgery again. Of the 75% UKA respondents, 23% had moderate to severe chronic pain, 86% were satisfied/very satisfied, and 88% would undergo the same surgery again.

**Conclusion:**

In Denmark, 25% of TKA patients and 23% of medial UKA patients experienced moderate to severe knee pain after 1 year. These numbers were higher than most previous estimates. Most patients were satisfied with the result of surgery and would undergo the same surgery again.

Knee arthroplasty is an effective treatment option for patients with knee osteoarthritis when non-surgical interventions are insufficient. While knee arthroplasty is generally safe and provides satisfactory results, some patients experience chronic pain after surgery [1,2]. Chronic postsurgical pain, defined as pain lasting over 3 months, has been associated with opioid dependence, reduced ability to work, and increased mortality [3,4]. Therefore, carefully weighing of potential benefits and risks is a substantial part of the decision to proceed with surgery. To support this process, updated evidence on the risk of adverse outcomes, including the incidence of chronic postsurgical pain, is essential.

Recently, a comprehensive review of long-term pain outcomes in unselected total knee arthroplasty (TKA) patients found that between 9.9% and 16% of patients had chronic pain at 12 months postoperatively [1]. The included studies were heterogeneous, had inconsistent results, and the review did not investigate patients undergoing unicompartmental knee arthroplasty (UKA). Moreover, the patients included in the review underwent surgery between 1993 and 2023. Since 1993, several advances in operative and perioperative care have been implemented, including fast-track surgery with multimodal analgesia, high-dose glucocorticoids, early mobilization, and markedly reduced hospital stays [5,6]. Also, medial UKA has gained popularity as an alternative to TKA in patients with isolated medial compartment arthritis [7]. It is unclear whether these improvements in perioperative care have affected the risk of chronic postsurgical pain [8]. Thus, there is a gap in knowledge regarding the current incidence of chronic pain after knee arthroplasty, particularly in patients undergoing UKA.

We aimed to investigate the incidence of chronic pain, pain characteristics, patterns of analgesic use, patient satisfaction, and willingness to undergo the same surgery again, 1 year after primary TKA and UKA.

## Methods

### Study design

This cross-sectional survey study was reported using the CROSS checklist for standardized reporting of survey studies (see Supplementary data 1) [9].

### Sample characteristics

For this survey, we included adult (18 years or older) patients undergoing primary TKA and medial UKA for primary osteoarthritis between August 1 and November 30, 2022. We excluded patients with bilateral surgery or unknown laterality of the operation within the 4-month sampling period. We also excluded patients who underwent surgery where additional components such as spacers or metaphyseal sleeves were used, because we suspected these were revision surgeries, incorrectly categorized as primary arthroplasties.

We included patients from all public and private hospitals performing knee arthroplasties in Denmark. In 2022, 27% of all primary knee arthroplasties were medial UKAs and Enhanced Recovery After Surgery protocols were widely implemented, resulting in a mean length of hospital stay of 1.2 days and 0.9 days for TKA and UKA patients, respectively [5].

### Data source

The Danish Health Data Authority identified eligible patients from the Danish National Patient Register, based on coded diagnosis and procedure [10]. Similarly, the Danish Clinical Quality Program diagnosis provided demographic and surgical data from the Danish Knee Arthroplasty Register [11]. The Danish Knee Arthroplasty Register has complete (100%) coverage and a yearly completeness of over 95% of all primary knee arthroplasties performed in Denmark [5]. The data in the National Patient Register and the Danish Knee Arthroplasty Register is entered separately, and both registries cover all public and private clinics performing TKA and UKA in Denmark. Only patients with identical diagnosis (i.e., primary knee osteoarthritis) and surgery (i.e., TKA or primary medial UKA) in the 2 registers were asked to participate.

### Data-collection methods

The survey comprised a cover letter (see Supplementary data 2) and a 22-item questionnaire (English translation and original Danish version: see Supplementary data 3 and 4). The cover letter briefly introduced the study and asked patients not to respond if they believed they had been contacted mistakenly. The author group drafted the questionnaire, which was then discussed and revised by a group of patients who had undergone hip or knee arthroplasty. All involved patient representatives approved the final version of the questionnaire. Below is a brief description and rationale for each question.

*Question 1* assessed overall satisfaction with surgery and was translated from the questionnaire used by the Swedish Arthroplasty Register to enable direct comparison [2]. Response options were very satisfied, satisfied, neither satisfied nor dissatisfied, dissatisfied, or very dissatisfied.

*Question 2* assessed willingness to undergo surgery again [12]. Response options were yes, no, or uncertain.

*Question 3* assessed how frequently the patients suffered from knee pain: “Do you still have pain in the operated knee?” [13]. Response options were yes—constantly, yes—daily, yes—a few times a week, yes—more rarely, or no. This question was mandatory for branching purposes, i.e., respondents who answered no automatically skipped questions 4–17.

*Question 4* was the 11-point numerical rating scale (NRS) score for knee pain, which is widely used and recommended [14,15]. A score of 0 represents no pain, whereas 10 represents worst pain imaginable. To facilitate comparison with other studies, we asked patients to rate their overall average pain in the last week [14].

*Questions 5–9* were the Western Ontario and McMaster Universities Osteoarthritis Index (WOMAC) pain domain (Likert scale, version 3.1) [16]. Patients were asked to rate their knee pain during activities such as standing upright or going up or down stairs during the last week. Response options were none, mild, moderate, severe, or extreme. The instrument is validated for osteoarthritis patients and recommended by the International Association for the Study of Pain (IMMPACT) [15].

*Question 10* assessed pain interference on daily life: “In total, how much does the pain in the operated knee bother you in your everyday life?” [13]. Response options were not at all, a little, some, much, or very much. Pain interference is often included in core outcome sets for clinical trials [15,17] and it is recommended to use disease-specific interference [15].

*Questions 11–17* were the Douleur Neuropathique 4 interview (DN4i), which assesses pain characteristics (burning, painful cold, and electric shocks) and associated symptoms (tingling, pins and needles, numbness, and itching) with yes/no questions [18,19]. We chose the DN4i over other neuropathic pain measures because it is widely used and validated, brief, and available in Danish (www.mapi-trust.org) [18].

*Question 18* was an open question asking if patients had other chronic pain conditions. We chose to use an open-text field to encourage reporting of all conditions. We believed this was more accurate than if patients categorized their pain condition from a list of options.

*Question 19* assessed patient-reported use of analgesics, including whether they were taken for pain in the treated knee or for other reasons. The type of analgesics was typed into an open text field to avoid limiting respondents to predefined options and to accommodate responses with potential typos.

*Questions 20–22* assessed height and weight, and asked for permission to contact them again later [20].

### Survey administration

On November 3, 2023, 12–16 months after surgery, a cover letter containing a unique survey link was sent to all patients via the encrypted governmental electronic letter system. This service is linked to the unique Civil Registration number, which ensures that only the intended recipient can access the mail. Survey responses were collected with the Research Electronic Data Capture (REDCap) software (https://project-redcap.org/). Non-respondents were sent reminders on November 23. We also planned to send text-message reminders to the non-respondents but could only obtain phone numbers of a subset of patients. Based on previous experience, we expected that sending text messages would result only in very few additional responses and therefore chose not to send them. To minimize errors, 2 authors (JL and SA) independently categorized the responses for questions with an open text field option (i.e., other pain conditions and analgesic use).

### Outcomes

*Primary outcome.* The primary outcome was the incidence of moderate to severe pain in the treated knee, defined as patients with ≥ 4 on a 0–10 NRS for average pain intensity in the treated knee during the last week [21]. We chose a dichotomized primary outcome as we believed this would be easier to interpret by patients and clinicians [22].

*Secondary outcomes.* The secondary outcomes were:

1. The proportion of patients who gave each response to each individual questionnaire item.
2. The distribution of NRS pain intensity scores for average pain intensity during the last week, reported as mean (standard deviation [SD]) and presented as a bar chart. The NRS score was reported only by the subgroup of patients who reported chronic postsurgical pain in question 3.
3. The median (interquartile range [IQR]) of the WOMAC pain domain total scores (0–20) and DN4i total scores (0–7).
4. The incidence of “possible” neuropathic pain, i.e., patients with a DN4i score of 3 or more [18,19].

### Data analyses

The sample size calculation was based on the TKA population. With an estimated 16% incidence of the primary outcome after TKA [2] and an anticipated response rate of 70% [23], a sample of 3,172 patients was required to achieve a 95% confidence interval (CI) with a margin of +1.5 percentage points (14.6–17.6). This level of certainty was deemed appropriate, as smaller differences may be difficult for clinicians and patients to interpret.

We handled the data using R (www.r-project.org). Categorical variables were reported as number of patients (percentage of all respondents). Continuous outcomes were reported as median (IQR). Generally, missing responses were not imputed. However, for the calculation of the WOMAC pain domain and DN4i total scores, we inserted the median of the non-missing values within the instrument. We did not plan to adjust for non-representativeness. However, we reported the baseline characteristics and surgical details of both respondents and non-respondents.

### Ethics, data sharing plan, funding, use of AI, and disclosures

This study was registered at the capital region of Denmark’s regional research listing with identifier P-2023-4, and the full protocol was uploaded at ClinicalTrials.gov on May 6, 2023 (NCT05900791). According to Danish legislation, approval from the national ethics committee is not needed for survey studies. Data is available from the corresponding author upon reasonable request. Moreover, the data will be stored with the Danish National Archives. This survey was part of a research program that also comprised a matching survey in total hip arthroplasty patients [24]. This work was supported by the Michaelsen foundation and the Danish Society of Anaesthesiology and Intensive Care Medicine’s research initiative. The funding sources had no influence on the planning, execution, or publication of this study. All authors declare no conflicts of interest in relation to the planning, conduct, and publication of this study. Complete disclosure of interest forms according to ICMJE are available on the article page, doi: 10.2340/17453674.2025.44898

## Results

### Patient characteristics

**Figure 1:**
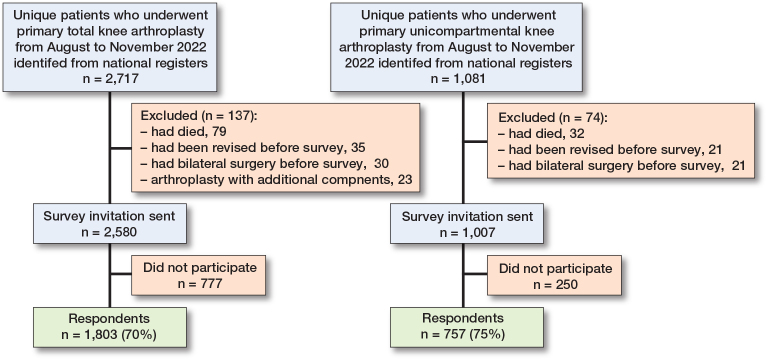
Participant flow chart.

70% of 2,580 eligible TKA patients and 75% of 1,007 eligible UKA patients responded (Figure 1). In both groups, the median time from surgery to response was 13 months (IQR 12–14). In the TKA group, respondents were slightly younger (mean difference 2 years, CI 1–3) and patients of female sex were less likely to respond (odds ratio 0.7, CI 0.6–0.9). All other demographic and surgical details were similar in TKA respondents and non-respondents (Table 1, see also Supplementary Table 1). UKA respondents and non-respondents had similar demographics and surgical details (Table 1, see also Supplementary Table 2).

**Table 1:** Characteristics of respondents and non-respondents

	TKA		UKA	
	Respondents (n = 1,803)	Non-respondents (n = 777)	Respondents (n = 757)	Non-respondents (n = 250)
Age, median (IQR)	71([64–76)	74 (64–79)	68 (62–75)	68 (61–78)
Women, n (%)	1,052 (58)	513 (66)	374 (49)	135 (54)
Body mass index, median (IQR)	29 (26–33)	29 (26–34)	28 ([26–32)	29 (26–33)
Disability, n (%)				
Unilateral osteoarthritis	742 (41)	308 (40)	402 (53)	135 (54)
Contralateral knee arthroplasty	483 (27)	227 (29)	154 (20)	53 (21)
Contralateral knee osteoarthritis	543 (30)	226 (29)	190 (25)	59 (24)
Other disabling condition	34 (1.9)	16 (2.1)	10 (1.3)	3 (1.2)
Previous surgery, n (%)				
None	1,519 (84)	687 (88)	649 (86)	211 (84)
Arthroscopic debridement	122 (6.8)	45 (5.8)	49 (6.5)	21 (8.4)
Cruciate ligament reconstruction	17 (0.9)	1 (0.1)	–	1 (0.4)
Femoral condyle osteosynthesis	4 (0.2)	–	–	–
Meniscectomy	111 (6.2)	35 (4.5)	57 (7.5)	16 (6.4)
Patella-stabilizing surgery	6 (0.3)	2 (0.3)	1 (0.1)	0 (0.0)
Proximal tibial osteotomy	9 (0.5)	1 (0.1)	–	–
Other	15 (0.8)	6 (0.8)	–	1 (0.4)
Duration of surgery, median minutes (IQR)	60 (53–70)	60 (51–70)	46 (38–55)	47 (40–56)
Anesthesia, n (%)				
General	1,223 (68)	543 (70)	488 (65)	167 (67)
Neuraxial	545 (30)	224 (29)	252 (33)	76 (31)
Combined	28 (1.6)	8 (1.0)	17 (2.2)	6 (2.4)
Nerve block alone	4 (0.2)	1 (0.1)	–	–
Pain catheter, n (%)	29 (1.6)	13 (1.7)	2 (0.3)	–
Local infiltration analgesia, n (%)	1,695 (94)	743 (96)	726 (96)	243 (98)

IQR: interquartile range; TKA: total knee arthroplasty; UKA: medial unicompartmental knee arthroplasty.

### Pain outcomes in TKA patients

**Figure 2:**
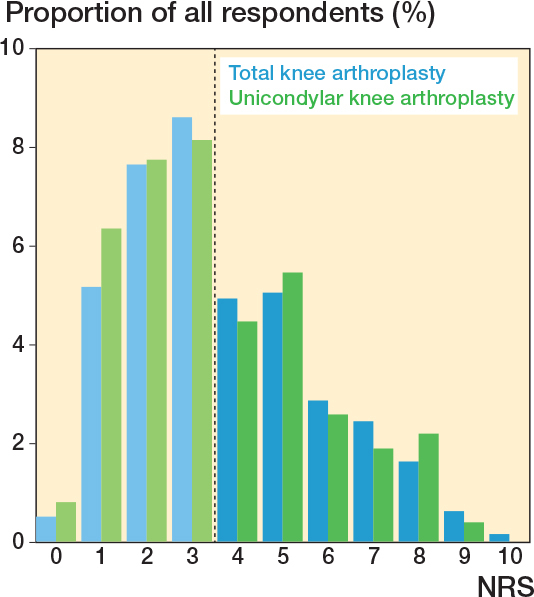
Distribution of 0–10 NRS pain intensity. “Please rate your pain in the operated knee by indicating the number that best describes your pain on average during the last week. 0 means ‘No pain’ and ‘10’ means ‘Pain as bad as you can imagine’.” The mean score was 3.7 (standard deviation [SD] 2.1) in TKA patients (n = 1,021) and 3.5 (SD 2.1) in UKA patients (n = 403). Note that the NRS question was not applied in respondents who reported no chronic postsurgical pain. Also note that 13 TKA patients and 8 UKA patients reported having knee pain but gave an NRS score of 0. Dotted vertical line depicts the threshold for moderate to severe pain (NRS ≥ 4).

Of the TKA respondents, 25% (CI 23–27) had moderate to severe chronic pain (NRS ≥ 4, Table 2). 43% of TKA patients were not surveyed regarding pain characteristics or severity as they reported no pain from the treated knee. The 57% of remaining patients reported a median NRS score of 3 (IQR 2–5) for average knee pain (Figure 2) and a median WOMAC pain domain score of 4 (IQR 2–7) (Table 3). In 20% of TKA patients, daily activities were somewhat, much, or very much affected by pain in the treated knee. “Possible neuropathic pain” was reported by 23% of TKA respondents (Table 4).

**Table 2:** Survey results. Numbers are the absolute number of respondents (percentage of all respondents)

	TKA (n = 1,803)	UKA (n = 757)
Persistent postsurgical pain		
NRS pain > 3	456 (25)	171 (23)
NA	–	2 (0.3)
Frequency of pain in the treated knee **^a^**		
Constantly	52 (2.9)	22 (2.9)
Daily	255 (14)	89 (12)
A few times a week	268 (15)	98 (13)
More rarely	446 (25)	196 (26)
No knee pain	782 (43)	352 (47)
Pain interference with everyday life		
Not at all	164 (9.1)	75 (9.9)
A little	481 (27)	197 (26)
Somewhat	242 (13)	83 (11)
Much	96 (5.3)	40 (5.3)
Very much	34 (1.9)	10 (1.3)
NA	4 (0.2)	–
Satisfaction		
Very satisfied	899 (50)	434 (57)
Satisfied	576 (32)	219 (29)
Neutral	181 (10)	52 (6.9)
Dissatisfied	109 (6.0)	38 (5.0)
Very dissatisfied	33 (1.8)	13 (1.7)
NA	5 (0.3)	1 (0.1)
Willing to undergo the same surgery again		
Yes	1,542 (86)	666 (88)
No	77 (4.3)	36 (4.8)
Unsure	133 (7.4)	37 (4.9)
NA	51 (2.8)	18 (2.4)

a The 782 pain-free patients were not asked further question regarding their knee pain, including the NRS.

TKA: total knee arthroplasty; UKA: medial unicompartmental knee arthroplasty; NRS: numerical rating scale (for average pain intensity in the treated knee during the last week); NA: not available.

**Table 3:** Western Ontario and McMaster Universities Osteoarthritis Index ((WOMAC) pain domain results. Numbers are the absolute number of respondents (percentage of all respondents) unless otherwise specified

	TKA (n = 1,803)	UKA (n = 757)
Knee pain going up or down stairs		
None (0)	128 (7.1)	59 (7.8)
Mild (1)	387 (22)	171 (23)
Moderate (2)	325 (18)	114 (15)
Severe (3)	117 (6.5)	38 (5.0)
Extreme (4)	26 (1.4)	12 (1.6)
NA	38 (2.1)	11 (1.5)
Knee pain walking on a flat surface		
None (0)	421 (23)	196 (26)
Mild (1)	329 (18)	110 (15)
Moderate (2)	179 (9.9)	66 (8.7)
Severe (3)	27 (1.5)	11 (1.5)
Extreme (4)	2 (0.1)	–
NA	63 (3.5)	22 (2.9)
Knee pain standing upright		
None (0)	282 (16)	142 (19)
Mild (1)	380 (21)	143 (19)
Moderate (2)	230 (13)	81 (11)
Severe (3)	58 (3.2)	12 (1.6)
Extreme (4)	6 (0.3)	1 (0.1)
NA	65 (3.6)	26 (3.4)
Knee pain sitting or lying		
None (0)	417 (23)	203 (27)
Mild (1)	346 (19)	117 (16)
Moderate (2)	160 (8.9)	50 (6.6)
Severe (3)	41 (2.3)	10 (1.3)
Extreme (4)	3 (0.2)	3 (0.4)
NA	54 (3.0)	22 (2.9)
Knee pain at night while in bed		
None (0)	460 (26)	212 (28)
Mild (1)	290 (16)	108 (14)
Moderate (2)	148 (8.2)	42 (5.5)
Severe (3)	54 (3.0)	19 (2.5)
Extreme (4)	14 (0.8)	7 (0.9)
NA	55 (3.1)	17 (2.2)
WOMAC pain domain **^a^**		
Total 0–20 score (IQR)	4 (2–7)	3 (2–6.5)
NA	2 (0.1)	–

a These values were calculated with missing values imputed as the median of the reported values within the instrument.

For abbreviations, see Table 2.

**Table 4:** Douleur Neuropathique 4 interview (DN4i) results. Numbers are the absolute number of respondents (percentage of all respondents) unless otherwise specified

	TKA (n = 1,803)	UKA (n = 757)
DN4i: Pain characteristics		
Burning	257 (14)	101 (13)
NA	144 (8.0)	64 (8.5)
Painful cold	156 (8.7)	52 (6.9)
NA	233 (13)	90 (12)
Electric shocks	168 (9.3)	87 (12)
NA	224 (12)	73 (9.6)
DN4i: Abnormal sensations		
Tingling	289 (16)	106 (14)
NA	229 (13)	82 (11)
Pins and needles	410 (23)	168 (22)
NA	177 (9.8)	55 (7.3)
Numbness	387 (22)	150 (20)
NA	185 (10)	70 (9.2)
Itching	139 (7.7)	45 (6)
NA	291 (16)	106 (14)
DN4i: Total score (0–7) **^a^**		
Total score, median (IQR)	2 (1–5)	2 (1–5)
NA	–	–
“Possible” neuropathic pain [18,19]		
DN4i score ≥ 3	411 (23)	160 (21)
NA	–	–

a These values were calculated with missing values imputed as the median of the reported values within the instrument.

For abbreviations, see Table 2.

Chronic pain from sites or conditions other than the treated knee was frequent (44%) in TKA patients, with most patients reporting back pain (16%) or pain from the other knee (14%) (see Supplementary Table 3). Fewer TKA patients took analgesics daily for chronic postsurgical knee pain (13%) than for coexisting chronic pain conditions (30%). Opioid use for chronic postsurgical knee pain was reported by 1.6% of TKA patients, while 5.4% reported opioid use for other reasons. Paracetamol and ibuprofen were the most used analgesics (see Supplementary Table 4).

### Pain outcomes in medial UKA patients

Of the UKA respondents, 23% (CI 20–26) had moderate to severe chronic postsurgical pain (see Table 2). 47% of UKA patients were not surveyed regarding pain characteristics or severity as they reported no pain from the treated knee. The remaining patients reported a median NRS score of 3 (IQR 2–5) for average knee pain during the last week (see Figure 2) and a median WOMAC pain domain score of 3 (IQR 2–6.5) (see Table 3). In 18% of UKA patients, daily activities were somewhat, much, or very much affected by pain in the treated knee. “Possible neuropathic pain” was reported by 21% of UKA respondents (see Table 4).

Chronic pain from sites or conditions other than the treated knee was frequent (39%) in UKA patients, with most patients reporting pain from the other knee (14%) or their back (14%) (see Supplementary Table 3). Fewer UKA patients took analgesics daily for chronic postsurgical knee pain (9.8%) than for coexisting chronic pain conditions (25%). Opioid use for chronic postsurgical knee pain was reported by 1.5% of UKA patients, while 4.0% reported opioid use for other reasons (see Supplementary Table 4).

### Satisfaction and willingness to undergo surgery again

Of all respondents, 82% of TKA patients and 86% of UKA patients were either satisfied or very satisfied with the result of surgery (see Table 2). Moreover, 86% of TKA patients and 88% of UKA patients would still have undergone surgery if they could go back in time.

## Discussion

We performed a nationwide survey on chronic postsurgical pain and patient satisfaction 1 year after primary TKA and UKA for osteoarthritis. We found that 25% of TKA patients and 23% of UKA patients reported moderate to severe chronic postsurgical pain. 82% of TKA patients and 86% of UKA patients reported being satisfied or very satisfied with the result of surgery.

A recent scoping review of long-term pain outcomes in TKA patients included 598,498 patients from 68 studies [1]. The authors found that 13% (9.9–16%) of patients had chronic pain at 12 months postoperatively. However, the definition of chronic pain varied between the included studies, the sample sizes were small, and the results were impacted by substantial loss to follow-up and risk of bias. Despite these limitations, it is surprising that in our study almost double the number of TKA patients had chronic postsurgical pain. Compared with our study, moderate–severe pain at 1 year was also less frequent in TKA patients registered in the Swedish Arthroplasty Register (18%), but this can probably be attributed to questionnaire differences [2]. For UKA patients, data on chronic postsurgical pain is much more limited. A Spanish study found that 22% of 64 UKA patients experienced moderate to severe pain (VAS ≥ 30) after 6 months, compared with only 17% of TKA patients [25]. In contrast, a comprehensive systematic review comparing TKA and UKA found that patients generally reported better outcomes, including pain, after UKA than after TKA [7]. In our study and in data from the Swedish Arthroplasty Register, patients who underwent UKA did not report substantially better long-term pain outcomes than patients who underwent TKA [2]. The main advantage of UKA compared with TKA may be superior early recovery rather than improved long-term pain outcomes [5,7].

Our reported satisfaction rates align with previously published data [2,26,27]. Using questions identical to those used by the Swedish Arthroplasty Register, we found very similar satisfaction levels for both TKA and UKA patients [2]. Notably, medial UKA accounted for less than 10% of knee arthroplasties in Sweden, compared with 27% in Denmark. Despite this disparity in utilization, the relative differences between TKA and UKA patients in terms of pain relief and satisfaction were similar across both countries.

In our study, we found a relatively high incidence of possible neuropathic pain. However, the diagnosis of definitive neuropathic pain is complex and requires physical examination and tests, and the true incidence of neuropathic pain in our sample is therefore uncertain [19]. Nonetheless, in a British sample, more than half of patients with chronic pain after TKA also reported possible neuropathic pain [28]. Together, this indicates that symptoms of neuropathic pain are present in a considerable proportion of patients, which should have implications for future research into treatment and preventive strategies for chronic postsurgical pain.

Our results are generalizable for patients undergoing TKA and UKA in a contemporary setting with high adherence to Enhanced Recovery After Surgery protocols [6]. The demographics of patients registered in the Danish Knee Arthroplasty Register have previously been shown to be similar to international data [29].

### Strengths

We sent the survey to all patients who underwent TKA and UKA in Denmark within the inclusion period, including those treated at private hospitals. Respondents and non-respondents were similar on almost all measured baseline variables, which strengthens the internal validity of the study. Finally, the study was prospectively registered, including predefined statistical methods and presentation of results.

### Limitations

The survey was cross-sectional and thus limited by having measured only a single postsurgical timepoint, and by missing baseline values. Further, patients who underwent revision surgery were not included. Although it is not recommended, unexplained pain is a relatively common reason for revision after TKA, and it is even more common following UKA [30,31]. Also, chronic postsurgical pain is more prevalent in patients who undergo revision surgery [32]. However, including revision cases would probably have little effect on the estimated incidence of chronic postsurgical pain, because only a small percentage of patients undergo revision surgery within 1 year: 1.6% for TKA and 2.2% for UKA [5].

We achieved relatively high response rates, with 70% of TKA patients and 75% of UKA patients responding. Nonetheless, the responses may not be missing at random, and this could therefore have impacted our findings. Moreover, the survey was powered to obtain a reasonably narrow confidence level in the estimate of patients with chronic pain after TKA and not UKA.

### Conclusion

1 year after primary surgery for primary osteoarthritis, 25% of TKA patients and 23% of medial UKA patients experienced moderate to severe pain. These numbers were higher than most previous estimates. Of TKA patients, 82% were satisfied or very satisfied with the outcome, and 86% would still have undergone surgery if they could go back in time. Of UKA patients, 86% were satisfied or very satisfied with the outcome, and 88% would undergo the same surgery again.

*In perspective,* the results presented provide clinicians and patients with important numbers for shared decision-making. Moreover, it is discouraging that although both surgery and the perioperative setting have evolved markedly, chronic postsurgical pain still seems to be a major issue in both TKA and UKA [5,6]. These findings should increase focus on prevention and treatment of chronic postsurgical pain, selection of patients for surgery, and modifiable risk factors [8,33].

### Supplementary data

Supplementary Data 1–4 and Supplementary Tables 1–4 are available on the article page, doi: 10.2340/17453674.2025.44898

## Supplementary Material



## Notes

JL and SO conceived the idea for the survey. JL drafted the protocol, sent surveys, performed data handling and data analysis, and drafted the manuscript. All authors took part in critically revising the protocol, wrote/revised the manuscript, and take responsibility for its intellectual content.

The authors would like to thank all patients who responded to the survey and the panel of patients who helped develop the questionnaire. Also, they wish to thank linguist Maud Signe Olsen who helped translate the questionnaire for the supplementary material.

Handling co-editor: Taco Gosens

*Acta* thanks Rudi Georg Bitsch and Siri Heijbel for help with peer review of this manuscript.
