# Supplementary material for: Chronic pain after primary total and medial unicompartmental knee arthroplasty for osteoarthritis: a Danish nationwide cross-sectional survey
Source: Acta Orthop. 2025 Oct 27;96:814–21. doi: 10.2340/17453674.2025.44898 (PMC12559960; doi:10.2340/17453674.2025.44898)
Supplement: Supplementary file 1 [file ActaO-96-44898-s1.pdf]

## Supplementary material

Laigaard J, Aljuboori SM, Nikolajsen L, Mathiesen O, Lunn TH, Lindberg-Larsen M, Overgaard S
Chronic pain after primary total and medial unicompartmental knee arthroplasty for osteoarthritis: a Danish nationwide cross-sectional survey. Doi: ****

# Contents

Supplementary material 2: Contact letter (Danish) .......... 2

Supplementary material 3: Questionnaire in English .......... 3

Supplementary material 4: Questionnaire in Danish .......... 5

Supplementary Table 1. Surgical characteristics of total knee arthroplasty respondents and non-respondents. Values are count (%) .......... 7

Supplementary Table 2. Surgical characteristics of unicompartmental knee arthroplasty respondents and non-respondents. Values are count (%) .......... 8

Supplementary Table 3. Survey results – self-reported other chronic pain sites/conditions. Values are respondents (%) .......... 9

Supplementary Table 4. Survey results – self-reported daily/almost daily use of individual analgesics. Values are respondents (%) .......... 11

## Supplementary material 2: Contact letter (Danish)

Region Hovedstaden

Kære borger

Vi skriver til dig, fordi det er registreret i landspatientregisteret, at du fik indsat et kunstigt knæ i [højre/venstre] side for omkring et år siden. Registret kan være behæftet med fejl, så hvis du ikke er opereret i knæet, bedes du se bort fra invitationen.

Vi beder dig besvare et kort spørgeskema som led i et forskningsprojekt. Formålet er at undersøge smerter og tilfredshed efter denne type operation. Svarene vil bl.a. blive brugt til at give fremtidige patienter bedre information om, hvad de kan forvente af operationen.

Spørgeskemaet tager få minutter at udfylde, og det er frivilligt at deltage. Brug linket herunder.

**[survey-link]**

Alle oplysninger vi får fra dig, vil blive behandlet fortroligt og vil ikke blive videregivet til andre uden for projektgruppen. Når resultaterne fremstilles, vil data være anonyme, så ingen vil kunne blive genkendt. Projektet er godkendt af Videnscenter for Dataanmeldelser, Region Hovedstaden.

Vi håber meget, at du vil besvare spørgeskemaet, uanset om du har smerter eller ej. På forhånd tak!

Med venlig hilsen

**Søren Overgaard**
Professor, overlæge, dr. med.
Ortopædkirurgisk afdeling
Bispebjerg Hospital

**Ole Mathiesen**
Professor, overlæge, ph.d.
Anæstesiologisk afdeling
Sjællands Universitetshospital
Køge

**Lone Nikolajsen**
Professor, overlæge, dr. med.
Bedøvelse og Operation samt
Intensiv afdeling
Aarhus Universitetshospital

**Martin Lindberg-Larsen**
Lektor, overlæge, ph.d.
Ortopædkirurgisk afdeling,
Odense Universitetshospital

Har du spørgsmål, eller er du kontaktet ved en fejl, bedes du kontakte læge og ph.d.-studerende Jens Holm Laigaard på Tlf. 4261 7377 (hverdage, kl. 9-14) eller Email jens.holm.laigaard@regionh.dk

## Supplementary material 3: Questionnaire in English

If you experience any issues responding to the survey, please contact Jens Holm Laigaard by telephone: 42 61 73 77 or email jens.holm.laigaard@regionh.dk

Satisfaction

1) How satisfied are you with the outcome of your knee replacement surgery?
   Very satisfied, Satisfied, Neither satisfied nor dissatisfied, Dissatisfied, Very dissatisfied

2) Knowing what your knee replacement surgery did for you, if you could go back in time, would you still have undergone this surgery?
   Yes, No, Uncertain

Pain in the operated knee

3) [Mandatory] Do you still have pain in the operated knee?
   Yes, constantly; Yes, daily; Yes, a few times a week; Yes, more rarely; No

4) [Mandatory if 1 ≠ ”No”] Please rate your pain in the operated knee by indicating the number that best describes your pain on average during the last week.
   0 means ‘No pain’ and ‘10’ means ‘Pain as bad as you can imagine’.

| | | | | | | | | | | |
|---|---|---|---|---|---|---|---|---|---|---|
| 0 | 1 | 2 | 3 | 4 | 5 | 6 | 7 | 8 | 9 | 10 |

What amount of knee pain have you experienced the last week during the following activities?

| | | | | | |
|---|---|---|---|---|---|
| 5) Walking on a flat surface | None | Mild | Moderate | Severe | Extreme |
| 6) Going up or down stairs | None | Mild | Moderate | Severe | Extreme |
| 7) At night while in bed | None | Mild | Moderate | Severe | Extreme |
| 8) Sitting or lying | None | Mild | Moderate | Severe | Extreme |
| 9) Standing upright | None | Mild | Moderate | Severe | Extreme |

Activities of daily living

10) In total, how much does the pain in the operated knee bother you in your everyday life?
   Not at all, A little, Some, Much, Very Much

Does the pain have one or more of the following characteristics?

| | | |
|---|---|---|
| 11) Burning | Yes | No |
| 12) Painful cold | Yes | No |
| 13) Electric shocks | Yes | No |

Is the pain associated with one or more of the following symptoms in the same area?

| | | |
|---|---|---|
| 14) Tingling | Yes | No |
| 15) Pins and needles | Yes | No |
| 16) Numbness | Yes | No |

| | | |
|---|---|---|
| 17) Itching | Yes | No |

Other pain

18) Do you have chronic pain, other than from your operated knee?
 Yes; No
 [If yes] Please describe your pain condition: _____

Analgesic use

19) Do you take analgesic medication(s) daily or almost daily?
 Yes; due to pain in the operated knee; Yes, due to other pain; No
 [If yes] Which medication(s)?: _____

Please insert your height and weight

20) [Mandatory] Weight: _____ (kg)
21) [Mandatory] Height: _____ (cm)

Contact

22) Can we contact you again regarding your responses?
 Yes; No
 [if yes] Please insert your contact information (telephone number, email address): _____

**Thank you so much for your participation. Your responses have been saved and you can exit the site.**
You are welcome to contact Jens Holm Laigaard by telephone 42 61 73 77 or email jens.holm.laigaard@regionh.dk if you have questions or comments regarding the survey.

## Supplementary material 4: Questionnaire in Danish

Oplever du problemer med at besvare spørgeskemaet, bedes du kontakte læge og phd-studerende Jens Holm Laigaard på tlf. 42 61 73 77 eller email jens.holm.laigaard@regionh.dk

Spørgsmålene handler om din [side] knæ, som du fik opereret for lidt over 1 år siden

Tilfredshed

1) Hvor tilfreds er du med resultatet af din knæprotese-operation?
   Meget tilfreds; Tilfreds; Hverken tilfreds eller utilfreds; Utilfreds; Meget utilfreds

2) Hvis du kunne gå tilbage i tiden, ville du stadig have taget imod operationen, når du nu ved hvad den har betydet for dig?
   Ja; Nej; Usikker

Smerter i det opererede knæ

3) [Mandatory] Har du fortsat smerter i det opererede knæ?
   Ja, konstant; Ja, hver dag; Ja, nogle gange om ugen; Ja, sjældnere; Nej

4) [Mandatory if 1 ≠ ”Nej”] Vurder venligst dine smerter i det opererede knæ ved at vælge det tal, som bedst beskriver den gennemsnitlige smerte i løbet af den seneste uge.
   0 betyder "ingen smerter" og 10 betyder "værst tænkelige smerter"

| | | | | | | | | | | |
|---|---|---|---|---|---|---|---|---|---|---|
| 0 | 1 | 2 | 3 | 4 | 5 | 6 | 7 | 8 | 9 | 10 |

Følgende spørgsmål handler om hvor mange smerter du har haft i knæet i løbet af den seneste uge. Angiv graden af smerter du har oplevet i følgende situationer?

| | | | | | |
|---|---|---|---|---|---|
| 5) Gå på jævnt underlag | Ingen | Lette | Moderate | Stærke | Meget stærke |
| 6) Gå op eller ned ad trapper | Ingen | Lette | Moderate | Stærke | Meget stærke |
| 7) Stående | Ingen | Lette | Moderate | Stærke | Meget stærke |
| 8) Sidde eller ligge | Ingen | Lette | Moderate | Stærke | Meget stærke |
| 9) Om natten, når du ligger ned (smerter, som forstyrrer din søvn) | Ingen | Lette | Moderate | Stærke | Meget stærke |

Dagligdags aktiviteter

10) Overordnet, hvor meget generer smerterne i det opererede knæ dig i din dagligdag?
    Slet ikke; Lidt; Noget; Meget; Rigtig meget

Har smerten et eller flere af følgende kendetegn?

| | | |
|---|---|---|
| 11) Brændende | Ja | Nej |
| 12) Smertefuld kold | Ja | Nej |
| 13) Elektriske stød | Ja | Nej |

Er smerten forbundet med et eller flere af følgende symptomer inden for samme område?

| | | |
|---|---|---|
| 14) Snurren | Ja | Nej |

| | | |
|---|---|---|
| 15) Stikken og prikken | Ja | Nej |
| 16) Følelsesløshed | Ja | Nej |
| 17) Kløe | Ja | Nej |

<u>Andre smertetilstande</u>
18) Har du kroniske smerter andre steder end i det opererede knæ?
Ja, Nej
[Hvis ja] Beskriv venligst din smertetilstand: _____ (Fx migræne, rygsmerter, fibromyalgi, smerter i modsatte knæ eller lign.)

<u>Behov for smertestillende medicin</u>
19) Tager du smertestillende medicin dagligt eller næsten dagligt?
Ja, pga. smerter i det opererede knæ; Ja, pga. andre smerter; Nej
[Hvis ja] Hvilke(n) medicin?: _____ (Fx panodil, tramadol, ipren, naturmedicin, morfin eller lign.)

<u>Indtast venligst din højde og vægt</u>
20) [Mandatory] Vægt: _____ (kg)
21) [Mandatory] Højde: _____ (cm)

<u>Kontakt</u>
22) Må vi kontakte dig igen, fx for at få uddybet dine svar?
Ja; Nej
[Hvis ja] Indtast venligst kontaktinformation (tlf.nummer, email-adresse): _____

**Tusind tak for din deltagelse. Din besvarelse er gemt, og du kan lukke siden.**
Har du spørgsmål eller kommentarer til spørgeskemaet, er du velkommen til at kontakte læge og phd-studerende Jens Holm Laigaard på tlf. 42 61 73 77 eller email jens.holm.laigaard@regionh.dk

**Supplementary Table 1. Surgical characteristics of total knee arthroplasty respondents and non-respondents. Values are count (%)**

| Total knee arthroplasty | TKA respondents (n = 1,803) | Non-respondents (n = 777) | P value |
|---|---|---|---|
| **Approach** | | | 0.7 |
| Medial parapatellar (through the quadriceps tendon) | 646 (36) | 263 (34) | |
| Medial parapatellar (vastus medialis split) | 47 (2.6) | 15 (1.9) | |
| Midline (through the quadriceps tendon) | 1,091 (61) | 491 (63) | |
| Midline (vastus medialis split) | 15 (0.8) | 6 (0.8) | |
| NA | 4 (0.2) | 2 (0.3) | |
| **Implant** | | | 0.99 |
| Attune CR | 92 (5.1) | 40 (5.1) | |
| NexGen CR | 105 (5.8) | 45 (5.8) | |
| Persona CR | 482 (27) | 206 (27) | |
| PFC Sigma CR | 637 (35) | 275 (35) | |
| Triathlon CR | 451 (25) | 195 (25) | |
| other | 16 (0.9) | 9 (1.2) | |
| NA | 20 (1.1) | 7 (0.9) | |
| **Cemented femoral component** | 1,081 (60) | 499 (64) | 0.1 |
| NA | 33 (1.8) | 13 (1.7) | |
| **Cemented tibial component** | 1,460 (81) | 649 (84) | 0.3 |
| NA | 33 (1.8) | 14 (1.8) | |
| **Patellar resurfacing** [a] | 1,238 (69) | 530 (68) | 0.3 |
| NA | - | 1 (0.1) | |

P values derive from Fischer's exact test (categorical variables).
[a] Proportion of all patients with a patellar component.
NA = not available; CR = cruciate-retaining

**Supplementary Table 2. Surgical characteristics of unicompartmental knee arthroplasty respondents and non-respondents. Values are count (%)**

| | UKA Respondents (n = 757) | Non-respondents (n = 250) | P value |
|---|---|---|---|
| **Approach** | | | 0.3 |
| Medial parapatellar (through the quadriceps tendon) | 198 (26) | 68 (27) | |
| Medial parapatellar (vastus medialis split) | 228 (30) | 59 (24) | |
| Midline (through the quadriceps tendon) | 61 (8.1) | 17 (6.8) | |
| Midline (vastus medialis split) | 2 (0.3) | 0 (0.0) | |
| Minimally invasive surgery | 265 (35) | 105 (42) | |
| NA | 3 (0.4) | 1 (0.4) | |
| **Implant** | | | 0.9 |
| NexGen Zuk | 36 (4.8) | 13 (5.2) | |
| Oxford | 641 (84.7) | 211 (84.4) | |
| other | 3 (0.4) | 2 (0.8) | |
| NA | 77 (10.2) | 24 (9.6) | |
| **Cemented femoral component** | 114 (15) | 36 (14) | 0.5 |
| NA | 15 (2.0) | 8 (3.2) | |
| **Cemented tibial component** | 115 (15) | 40 (16) | 0.6 |
| NA | 16 (2.1) | 8 (3.2) | |

P values derive from Fischer's exact test (categorical variables).
NA = not available

**Supplementary Table 3. Survey results – self-reported other chronic pain sites/conditions. Values are respondents (%)**

| | TKA respondents (n = 1,803) | UKA respondents (n = 757) |
|---|---|---|
| **Any other chronic pain condition** | **791 (44)** | **296 (39)** |
| **Head or neck** | **61 (3.4)** | **30 (4.0)** |
| Headache | 25 (1.4) | 13 (1.7) |
| Neck | 43 (2.4) | 20 (2.6) |
| **Upper extremities** | **117 (6.5)** | **49 (6.5)** |
| Arm | 9 (0.5) | 4 (0.5) |
| Clavicle | 1 (0.1) | 1 (0.1) |
| Elbow | 2 (0.1) | 1 (0.1) |
| Hands | 46 (2.6) | 18 (2.4) |
| Shoulder | 67 (3.7) | 33 (4.4) |
| Wrist | 11 (0.6) | 2 (0.3) |
| **Back** | **289 (16)** | **104 (14)** |
| Back | 245 (14) | 94 (12) |
| Lower back | 48 (2.7) | 12 (1.6) |
| **Chest and abdomen** | **3 (0.2)** | **4 (0.5)** |
| Abdomen | 1 (0.1) | 3 (0.4) |
| Chest | 2 (0.1) | 1 (0.1) |
| Groin | 5 (0.3) | 2 (0.3) |
| **Lower extremities** | **391 (22)** | **154 (20)** |
| Ankle | 28 (1.6) | 13 (1.7) |
| Buttock/thigh | 6 (0.3) | 6 (0.8) |
| Claudicatio intermittens | 1 (0.1) | - |
| Contralateral knee | 258 (14) | 107 (14) |
| Feet | 49 (2.7) | 17 (2.2) |
| Hip | 72 (4.0) | 36 (4.8) |
| Leg | 25 (1.4) | 8 (1.1) |
| Sciatica | 4 (0.2) | - |
| **Other** | **94 (5.2)** | **36 (4.8)** |
| Arthritis urica (unspecified site) | 2 (0.1) | - |
| Fibromyalgia (unspecified site) | 15 (0.8) | 12 (1.6) |
| Inflammatory arthritis (unspecified site) | 10 (0.6) | 1 (0.1) |
| Peripheral neuropathy | 11 (0.6) | 5 (0.7) |
| Phantom limb pain | 1 (0.1) | - |
| Polymyalgia rheumatica (unspecified site) | 8 (0.4) | - |
| Other/ambiguous | 50 (2.8) | 18 (2.4) |
| **No site/condition stated** [a] | **84 (4.7)** | **24 (3.2)** |
| **Not available** [b] | **43 (2.4)** | **9 (1.2)** |

*Question: Do you have chronic pain, other than from your operated knee? Yes; No. [If yes] Please describe your pain condition: _____*

[a] Patients who reported having another chronic pain condition but did not fill in where or which condition.
[b] Respondents who skipped this question.
TKA = total knee arthroplasty; UKA = medial unicompartmental knee arthroplasty.

## Supplementary Table 4. Survey results – self-reported daily/almost daily use of individual analgesics. Values are respondents (%)

| | TKA respondents (n = 1,803) | | UKA respondents (n = 757) | |
|---|---|---|---|---|
| | For other pain condition | For postsurgical knee pain | For other pain condition | For postsurgical hip pain |
| **Not available [a]** | **43 (2.4)** | | **9 (1.2)** | |
| **Any analgesic** | **549 (30)** | **228 (13)** | **192 (25)** | **74 (9.8)** |
| **Any opioid** | **97 (5.4)** | **28 (1.6)** | **30 (4.0)** | **11 (1.5)** |
| Buprenorphine | 1 (0.1) | - | 1 (0.1) | - |
| Codeine | 8 (0.4) | 4 (0.2) | 4 (0.5) | 2 (0.3) |
| Fentanyl | 4 (0.2) | - | - | - |
| Methadone | 2 (0.1) | - | - | - |
| Morphine | 36 (2.0) | 12 (0.7) | 19 (2.5) | 8 (1.1) |
| Oxycodone | 9 (0.5) | 3 (0.2) | 1 (0.1) | 1 (0.1) |
| Tramadol | 43 (2.4) | 9 (0.5) | 6 (0.8) | - |
| **Any NSAID** | **153 (8.5)** | **67 (3.7)** | **61 (8.1)** | **26 (3.4)** |
| Aspirin | 6 (0.3) | 1 (0.1) | 2 (0.3) | 1 (0.1) |
| Celecoxib | 1 (0.1) | - | | |
| Dexibuprofen | 1 (0.1) | - | 1 (0.1) | - |
| Diclofenac | 3 (0.2) | 2 (0.1) | 1 (0.1) | 1 (0.1) |
| Etodolac | 2 (0.1) | - | | |
| Ibuprofen | 137 (7.6) | 65 (3.6) | 57 (7.5) | 21 (2.8) |
| Naproxen | 5 (0.3) | - | 2 (0.3) | 1 (0.1) |
| Topical NSAID | | | 0 (0.0) | 2 (0.3) |
| **Any antidepressant (for pain)** | **8 (0.4)** | **1 (0.1)** | **3 (0.4)** | **-** |
| Amitriptyline | 5 (0.3) | - | 1 (0.1) | - |
| Duloxetine | 2 (0.1) | 1 (0.1) | 2 (0.3) | - |
| Nortriptyline | 1 (0.1) | - | | |
| **Any anticonvulsant (for pain)** | **31 (1.7)** | **8 (0.4)** | **15 (2.0)** | **2 (0.3)** |
| Carbamazepine | - | - | 1 (0.1) | - |
| Gabapentin | 22 (1.2) | 7 (0.4) | 12 (1.6) | 2 (0.3) |
| Lamotrigine | 1 (0.1) | - | | |
| Oxcarbazepine | 1 (0.1) | - | | |
| Pregabalin | 8 (0.4) | 1 (0.1) | 3 (0.4) | - |
| **Any muscle relaxant (for pain)** | **1 (0.1)** | **-** | **2 (0.3)** | **1 (0.1)** |
| Baclofen | 1 (0.1) | - | 1 (0.1) | - |
| Chlorzoxazone | 2 (0.1) | 1 (0.1) | 1 (0.1) | 1 (0.1) |
| **Any other** | **439 (24)** | **188 (10)** | **151 (20)** | **62 (8.2)** |
| Low-dose naltrexone | 3 (0.2) | - | | |
| Natural medicines | 4 (0.2) | 1 (0.1) | 3 (0.4) | - |
| Paracetamol | 438 (24) | 188 (10) | 151 (19.9) | 62 (8.2) |
| **Not stated [a]** | **55 (3.1)** | **24 (1.3)** | **25 (3.3)** | **9 (1.2)** |

*Question: Do you take analgesic medication(s) daily or almost daily? Yes; due to pain in the operated knee; Yes, due to other pain; No. [If yes] Which medication(s)?: _____*

[a] Patients who reported daily/almost daily analgesic use but did not fill in which analgesics.

17 (0.9%) of 1,803 respondents skipped this question (question 19). NSAID = nonsteroidal anti-inflammatory drug.
